# Supplementary material for: Biphenylene: A Two−Dimensional Graphene−Based Coating with Superior Anti−Corrosion Performance
Source: Materials (Basel). 2022 Aug 18;15(16):5675. doi: 10.3390/ma15165675 (PMC9414146; doi:10.3390/ma15165675)
Supplement: Supplementary file 1 [file materials-15-05675-s001.zip › materials-1833251-supplementary.pdf]

Supporting Information for

Biphenylene: a 2D graphene-based coating with super anti-corrosion performance

S1. The POSCAR file of biphenylene

```
biphenylene
   1.00000000000000
     3.7630211888154195    0.0000000000000000    0.0000000000000000
     0.0000000000000000    4.5124186190957802    0.0000000000000000
     0.0000000000000000    0.0000000000000000   15.0000000000000000
   C
     6
Direct
  0.3496775938954855  0.0289839577403512  0.5000000000000000
  0.6557611870230957  0.2073437911059131  0.5000000000000000
  0.6558365672167312  0.5293675429269840  0.5000000000000000
  0.3497236524059630  0.7077204008716791  0.5000000000000000
  0.0436175124324336  0.5293806201848739  0.5000000000000000
  0.0435634280262889  0.2073337441701852  0.5000000000000000

  0.00000000E+00  0.00000000E+00  0.00000000E+00
  0.00000000E+00  0.00000000E+00  0.00000000E+00
  0.00000000E+00  0.00000000E+00  0.00000000E+00
```

0.00000000E+00	0.00000000E+00	0.00000000E+00
0.00000000E+00	0.00000000E+00	0.00000000E+00
0.00000000E+00	0.00000000E+00	0.00000000E+00

S2. The origin of band structure and DOS spectra of Biphenylene

The results of the electronic structure calculations, i.e., the band structure, total density of states (TDOS) and partial DOS (PDOS) of the biphenylene monolayer are presented in Figure S1 (a,b). Previous studies have demonstrated that the catalytic activity of many 2D materials is closely associated with their electronic structures [1,2]. To obtain a preliminary under standing of the activity of 2D biphenylene, we investigated its electronic properties by computing the band structure. 2D biphenylene exhibits a metallic property with two energy bands crossing the Fermi level (Figure S1 a), which is different from that of semi-metallic graphene. According to the density of states (DOS) calculations (Figure S1 b), the noteworthy part of this DOS is near the Fermi level, where the atom of C_2 is in full effect, contrary to C_1 which gives a very little contribution. In the shape of PDOS, the s, p_x, p_y, and p_z orbitals are given separately. PDOS of C_1 and C_2 show striking resemblance in some sections, but are wildly different in other areas. Both atoms show a heavy involvement of p_z-orbitals near the Fermi level; while, as mentioned before, only C_2 is intensely contributing when it gets close to the line, which could be further confirmed by partial charge density analysis. Overall, these abundant electronic states could accelerate electron transfer and improve the binding to adsorbates, which is desirable for electrocatalysis.

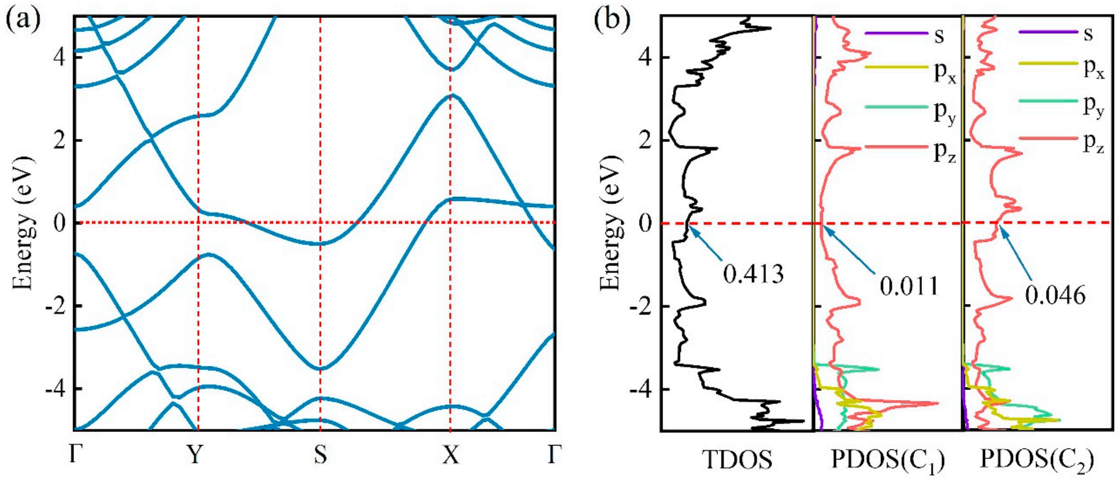

Figure S1. (a) The band structure of biphenylene. (b) The DOS of biphenylene.
